# The Guide Board

**Published:** 1870-11

**Authors:** 


### "THE GUIDE BOARD"

Is the title of a book of our writing, published by subscription only, by D. E. Fisk & Co., of Springfield, Mass., corner of Main and Bridge Streets, to be had of them or Martin & McKinney, 1308 Chesnut Street, Philadelphia, Penn. 752 pp. 8 vo., elegantly bound in muslin, for $4. It is not to be had at any bookstore; it is also bound in various styles in leather, which is more enduring, at $4.50, $5.50, $6.00 and $7.00. It is not often that an author criticises his own writings; but we speak of this book in the interest of our common humanity as one which, if placed in every intelligent family wherever the English language is spoken, would have an incalculable influence for good in the promotion of human health, because it tells plainly what every reflecting person wants to know about eating, drinking, sleeping, exercise, clothing; how to follow any of the occupations of life; how to live in any part of the world, and yet keep well. Men and women live to old age in all countries and in all climes—in all occupations, pursuits and callings—and it is believed what one can do, all can do; and how to do it, is pointed out so clearly and in so few words, that the commonest reader may understand, and the busiest man may find time to read. There is perhaps not a foreign, not an un-English word in the whole book, and very few indeed that a child of a dozen years could not understand without looking into a dictionary. Over four hundred different subjects are treated pertaining to physical, mental, and moral health.

### FARMERS.

There is a large amount of valuable information for farmers, in reference to overtaxing farmers' wives—to dwellings, barns, stables, ice-houses, and to their most healthful locations. There is also a large amount of

### FAMILY READING, suitable for boys and girls, about domestic duties, cooking, baking bread, the teeth, the feet, the hands, the finger-nails, the hair, eye-sight, hearing, bathing, boating, gymnastics, plays and games; about keeping tidy rooms, dressing neatly; illustrations of the value of promptitude, punctuality, perseverance, politeness, generosity, good nature; the dreadful nature and effects of going in debt; how health is lost every year by multitudes of the young, which could have been avoided if they had a little more information on the subject; it shows in many ways, and in various places, how dyspepsia and consumption could be avoided. There is much said about rearing children, school dangers, rash punishments, when and what kind of persons to marry, marriage and married life, and, in short, how to live so as to attain a cheery, happy old age. It is a book which can be read with amusement and profit the year round, and for a succession of years together; for there is such a variety of subjects treated, that a day cannot pass in any average family wherein the "Guide Board" could not be consulted with profit; because the things spoken of pertain to the every-day affairs of life, and will be as true years hence as to-day. It is a book which may well be handed down from father to son; hence all are advised to purchase the plain, substantial, leather-bound volume. The reader will please remember that almost everything in the book, of true practical interest, was learned from others, or from personal observation in the course of near forty years' practice of medicine. We bespeak for the energetic, enterprising and liberal publishers, a permanently increasing call for the book, on which they have fearlessly yet confidently expended a large amount of money, relying upon the intelligence of the people for a renumerative patronage. Several thousand volumes have been already sold, and other editions are rapidly following. The engraving of the author was taken on the blessed side of fifty.

